# Subdural Empyema in Children

**DOI:** 10.5539/gjhs.v5n6p54

**Published:** 2013-08-14

**Authors:** Mohamed A. Hendaus

**Affiliations:** 1 Department of Pediatrics, Hamad Medical Corporation, Doha, Qatar

**Keywords:** subdural empyema, children, meningitis

## Abstract

Subdural Empyema in infants and children might be life threatening if not managed properly. A search of the Pub Med database was carried out using a combination of the following terms: Subdural empyema, children, and management. Neurosurgical textbooks were reviewed as well. The prevalence, etiology, clinical features, investigations and management of SDE are reviewed in this article. Conservative management with antibiotics and follow up imaging is recommended if there are no focal deficits, change in mental status or if the patient is responding well to antibiotics. Alternatively, craniotomy is warranted in addition to antibiotics therapy. The surgeon might opt for burr holes in case the patient is frail or in septic shock.

## 1. Introduction

Subdural Empyema (SDE) is usually referred to an intracranial purulent material between the dura and arachnoid matter (Sucu, Koksal, Aksoy, Aydin, & Caylan, 2006). In infants and young children, SDE could be life threatening if not managed properly (Liu, Chen, Tu, Lee, & Wu, 2010). The mortality rate of patients with SDE is around 4%, while the morbidity for survivors is even higher with residual neurologic deficits reaching up to 50%, hemiparesis 15-35% and persistent seizures 12-37.5% (Germiller & Sparano, 2006).

### 1.1 Prevalence and Sex

SDE develops in 39-60% of patients with pyogenic meningitis, but only 1-2% in patients with bacterial meningitis (Liu et al., 2010). SDEs constitute 15-25% of pyogenic intracranial infections (Lefebvre, Metellus, Dufour, & Bruder, 2009), and are more common in males (62% compared to females) (De Bonis et al., 2009).

### 1.2 Location

SDE are categorized anatomically to supra-tentorial, infra-tentorial or affecting the spinal canal (Ramamurthi & Panole, 2007). Review of the literature shows that SDE can occur anywhere in the subdural space, but the majority is in the supratentorial compartment. In addition, it is wise to be aware that SDEs might present with associated lesions like soft tissue infection, extradural collection, intra-axial abscess or bone infection (Farah, Kandasamy, May, Buxton, & Mallucci, 2009).

### 1.3 Etiology

Meningitis is the most common cause of SDE in infants (Liu et al., 2010). However, in older children, sinusitis and otitis media are the most common sources (Agrawal, Timothy, Pandit, Shetty, & Shetty, 2007). If the source of SDE is sinusitis, then the frontal sinus is the most common culprit followed by the ethmoid, sphenoid, and maxillary sinuses (Waseem, Khan, & Bomann, 2008). The infection is spread from the paranasal sinuses to the subdural space through bone erosion or takes the hematogenous route (Farah et al., 2009). It has been reported that lumbar puncture might lead to spinal SDE (Ramamurthi & Panole, 2007). Other causes of SDE are iatrogenic such as subdural hematoma drainage, craniotomy and intracranial pressure monitoring (Kapu, Pande, Vasudevan, & Ramamurthi, 2013).

### 1.4 Clinical Features

Infants and young children with SDE might present with altered mental status, meningeal irritation, and/or signs and symptoms of intracranial pressure. Some studies showed that 40% of patients with SDE present with seizures (De Bonis et al., 2009).

Patients with SDE due to sinusitis have a wide range of clinical presentation. They can present with the typical symptoms of sinusitis like fever, headache and purulent rhinorrhea; in addition, tearing of the eyes, photophobia and painful parathesias in the area covered by the trigeminal nerve. Overall, patients with SDE due to frontal sinusitis have more subtle signs and symptoms compared to infection of other sinuses (Waseem et al., 2008).

Signs and symptoms of SDE due to sinusitis can precede the disease anywhere between 12 days and six weeks prior to the diagnosis. Since SDE can be associated with extracranial manifestation (37%), it is wise to look for peri-orbital edema, proptosis, facial swelling, diplopia or pain when moving the extraocular muscles (Bruner, Littlejohn, & Pritchard, 2012).

### 1.5 Microbiology

There are many pathogens that can cause SDE and it depends on the route of the infection as well as the age of the patient. *Enterobacteriacee, Group B streptococci* or *Listeria monocytogenes* are usually the cause of SDE in neonates with meningitis; while in children with SDE due to meningitis, the pathogens are: *H. influenza, Escherichia coli, S. pneumoniae or Neisseria meningitides*. Pathogens from the paranasal sinuses are more numerous e.g. *Alpha-hemolytic streptococci*, anaerobic *streptococci*, non-hemolytic *streptococci, S. aureus*, *Bacteroides* species and *Enterobacteriaceae* (Agrawal et al., 2007). *Streptococcus milleri* has been reported recently as the most common organism of SDE due to sinusitis (Waseem et al., 2008). If the source of SDE is otitis media, then the responsible pathogens are alpha-hemolytic *streptococci, P. aureginosa, Bacteroides* species and *S. aureus*. It is also worth mentioning that *S. aureus, S. epidermidis*, and *Enterobacteriaceae* are acquired during trauma and might lead to SDE (Agrawal et al., 2007).

Wu et al studied the micro-organisms in 31 pediatric patients with SDE and they found that the most common pathogen was *Streptococcus pneumoniae* (16.1%), followed by *group B Streptococcus* (12.9%), *Haemophilus influenzae type b* (12.9%), *Salmonella spp*. (12.9%), *Escherichia coli* (9.7%) and *Pseudomonas aeruginosa* (9.7%). The authors also stated that only 3 out of the 31 patients had prior otorhinolaryngeal infections (Wu, Chiu, & Huang, 2008). In addition, *Mycobacterium tuberculosis* SDE has been reported (Vijayakumar, Sarin, & Mohan, 2012).

SDE can be poly-microbial most commonly *streptococci* and anaerobe bacteria. However, there are reports of co-infection of *Streptococcus intermedius* with *Streptococcus pneumonia* (Greve, Clemmensen, Ridderberg, Pedersen, & Møller, 2011), and *Streptococcus constellatus* with *Actinomyces viscosus* (Bouziri, Khaldi, Smaoui, Menif, & Ben Jaballaha, 2011).

## 2. Investigations

### 2.1 Blood

C-reactive protein, erythrocyte sedimentation rate, and white blood cell count are usually elevated in patients with SDE. In addition, it has been reported that diabetes or hyperglycemia are risk factors for intracranial empyemas (Adame, Hedlund, & Byington, 2005).

### 2.2 Cerebrospinal Fluid

Bacterial meningitis is considered a major source of SDE in infants and CSF culture is the “gold standard” for the diagnosis. Ancillary tests like PCR, latex agglutination test, and gram stain are helpful for the diagnosis of bacterial meningitis, especially for patients who were treated with antibiotics prior to the CSF sampling (Brouwer, Tunkel, & Van de Beek, 2010).

Since latex agglutination test results are usually ready in a short period of time, it could an indispensible tool for rapid identification of the micro-organism and hence proper treatment (Surinder, Bineeta, & Megha, 2007).

### 2.3 Imaging

Imaging of the head is recommended for every patient suspected of having SDE.

Cranial ultrasonography is usually the first imaging mode to be ordered in infants because it is safe, cost-effective, and it usually differentiates subdural empyema from subdural effusion (Liu et al., 2010).

Sometimes a skull radiograph can be useful to look for skull fracture, osteomyelitis or a lodged foreign body (Ramamurthi & Panole, 2007).

Computed Tomography (CT) of the head is considered cost-effective and accessible. However, it can be normal in up to 50% in patients with SDE (Gupta et al., 2011). When using CT as an imaging modality, SDE will appear crescentic in shape over the cerebral convexity with a surrounding rim that is enhanced with the use of contrast (Waseem et al., 2008).

**Figure 1:**
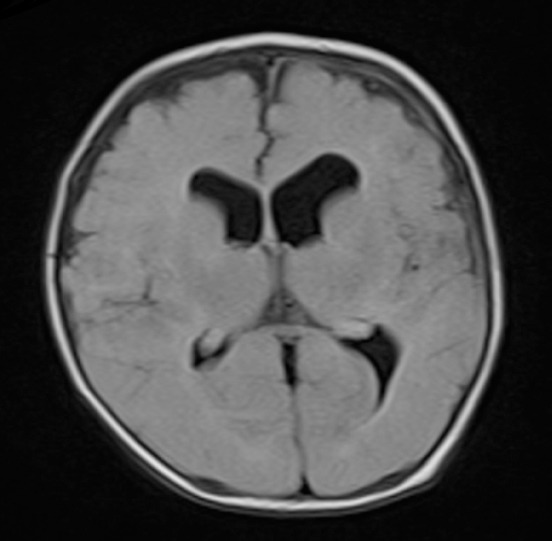
T2-Dark fluid MRI showing left temporal extra-axial collection (10 mm) in a newborn

Magnetic resonance imaging (MRI) has a sensitivity of 93% (Bruner et al., 2012), and is considered the best imaging mode for SDE because it usually portrays clearly the collections, and reveals signs of meningeal infections (Figure 1). In addition, diffusion-weighted imaging (DWI) ameliorates diagnostic preciseness and assists in monitoring of antibiotic therapy (Aldinger et al., 2013).

However, MRI machines are not available in every health care setting and it could delay the urgent interventions (Gupta et al., 2011).

## 3. Management

### 3.1 Conservative

Conservative treatment is recommended for patients with non-focal neurological deficits, no changes in mental status, empyema is limited and localized except the posterior fossa, and if the response to antibiotics is adequate. However, following the conservative approach will require frequent imaging to follow up on the SDE (De Bonis et al., 2009).

Another school that advocates conservative management indicates that patients who respond well clinically as well as radiographically, antibiotics alone suffice; otherwise neurosurgical intervention is warranted (Wanna et al., 2010).

The antibiotics regimen should be chosen per route of infection; thereafter, narrow the coverage per culture identification and sensitivity (Liu et al., 2010). Due to the lack of large randomized studies for the treatment of SDEs, antibiotic regimens are extrapolated from large studies of brain abscess management (De Bonis et al., 2009).

If the organism is unknown, then oxacillin plus ceftriaxone/cefotaxime plus metronidazole is recommended; however, if there is a suspicion for methicillin-resistant S. aureus, then vancomycin instead of oxacillin is warranted (De Bonis et al., 2009). Linezolid is an alternative treatment in case of conventional antibiotic regimen failure (Lefebvre et al., 2009).

As far as the duration of antibiotic treatment, it differs among practices. For instance one of the practices recommends at least two weeks through the intravenous route, followed by six weeks of oral therapy. If osteomyelitis concurs with SDE, then the oral route is usually eight weeks (De Bonis et al., 2009). The second practice states that intravenous route should be for six weeks followed by an oral course of four to six weeks (Kurschel, Mohia, & Weigl, 2006).

Intravenous steroids use has been advocated due to its function in reducing edema, swelling and inflammation (Lefebvre et al., 2009).

Depending on the presentation, lowering intracranial pressure might be required with modalities like elevation of the head, mannitol or ventriculostomy (Bruner et al., 2012).

Anti-seizure medications are recommended as prophylaxis because of the high rate of seizures associated with SDE (Bruner et al., 2012). Regarding the duration of therapy, there are different schools: some advocate for the treatment only during the acute phase of disease, others prefer indefinitely and the rest prefer to discontinue the anticonvulsants once the patient is seizure free for at least two years (De Bonis et al., 2009).

### 3.2 Surgical

The two most surgical procedures that have been conducted for SDE are craniotomy and burr holes. There is a consensus that Burr hole compared to craniotomy has a higher rate of SDE recurrence (Ramamurthi & Panole, 2007; Madhugiri et al, 2011; Legrand et al., 2009; Nathoo, Nadvi, Gouws, & van Dellen, 2001; Banerjee, Pandey, Devi, Sampath & Chandramouli, 2009; Mat Nayan, Mhd Haspani, Abd Latif, Abdullah, & Abdullah, 2009).

However, one study showed that there was no difference between the two modalities in terms of recurrence rate, antibiotic therapy duration, neurological outcome and complications (Liu et al., 2010).

Craniotomy is considered as the technique of choice because it allows complete evacuation of the empyema, and it decompresses the underlying cerebral hemisphere (Nathoo et al., 2001). However, bridging veins injury might be a complication of craniotomy (Kapu et al., 2013).

There are situations were burr holes are recommended over craniotomy like patients with septic shock or with parafalcine empyemas (Nathoo et al., 2001). Other indications for burr holes use include emergency situations or if the patient is considered frail (Ramamurthi & Panole, 2007).

The disadvantages of Burr Hole are that the technique is not optimal for multi-loculated subdural collections and that can lead to secondary injury of the cortex and therefore might exacerbate infections (Nathoo et al., 2001).

In terms of surgical intervention, there is a consensus that the mortality rate is 28% in patients with SDE, 23.3 % if burr holes technique is used and 8.4 to 11.5 % if craniotomy or craniectomy is performed (Ramamurthi & Panole, 2007).

Some surgeons use functional endoscopic sinus surgery (FESS) since it might aid in drainage and recovery (Bruner et al., 2012), and others conduct percutaneous needle aspiration of SDE via the fontanelle in infants (Ramamurthi & Panole, 2007).

In recent studies, specialists have been supporting the idea of Hollow screws for the diagnosis and treatment of SDE especially when CT and MRI studies are inconclusive (Aldinger et al., 2013).

### 3.3 Hyperbaric Oxygen Therapy

Hyperbaric oxygen therapy (HBO) has been an option for the treatment of brain abscess in children. The rationale behind HBO is the induction of hyperoxia in damaged tissues and hence will lead to restoration of the white blood cells functions. However, this treatment can have complications like barotraumatic lesions to the middle ear, inner ear, nasal sinuses and lung, oxygen toxicity, myopia and cataracts (Kurschel et al., 2006).

## 4. Follow-Up and Prognosis

Neurological deficits and cerebral herniation on CT scan are indicators of poor prognosis in patients with SDE (Legrand et al., 2009).

Nowadays, the survival rate for children with SDE is more than 90% if surgical intervention was done on timely basis (Ramamurthi & Panole, 2007).

Rate of disability due to SDE depends of the timing of the surgical intervention (if needed). If the intervention is done within 72 hours of symptoms the chance of disability is 10%, while the probability increases to 70% if the surgery is conducted after 72 hours. The health care provider has to have an ethical responsibility in counseling patients to be compliant with the antibiotics as well as with the anticonvulsants (Agrawal et al., 2007).

## 5. Conclusion

Subdural Empyema is a life threatening entity if not diagnosed early. Conservative management with antibiotics and follow up imaging is recommended if there are no focal deficits, change in mental status or if the patient is responding well to antibiotics. Alternatively, craniotomy is warranted in addition to antibiotics therapy. The surgeon might opt for burr holes in case the patient is frail or in septic shock.
